# The association between obesity and dengue virus (DENV) infection in hospitalised patients

**DOI:** 10.1371/journal.pone.0200698

**Published:** 2018-07-17

**Authors:** Victoria Phooi Khei Tan, Chin Fang Ngim, Erika Ziyan Lee, Amutha Ramadas, Lian Yih Pong, Joo Ing Ng, Sharifah Syed Hassan, Xuan Ye Ng, Amreeta Dhanoa

**Affiliations:** 1 Jeffrey Cheah School of Medicine and Health Sciences, Monash University Malaysia, Bandar Sunway, Selangor, Malaysia; 2 Clinical School Johor Bahru, Jeffrey Cheah School of Medicine and Health Sciences, Monash University Malaysia, Johor Bahru, Johor, Malaysia; University of Malaya, MALAYSIA

## Abstract

Both obesity and DENV infections are growing public health concerns that have far-ranging socioeconomic effects, especially in developing countries. Despite the increasing prevalence of these conditions, there is a scarcity of data investigating the potential relationships between these two entities. Our study aims to examine the influence of obesity on various clinical and laboratory parameters amongst patients with DENV infections. A total of 335 hospitalized patients aged >12 years who were DENV non-structural protein 1 (NS1) antigen-positive were enrolled in this study. Clinical and laboratory variables were compared between patients with and without obesity. Multivariate analysis showed that the following admission clinical findings and laboratory results were independently associated with obesity; chills and rigors (AOR:2.653, 95% CI: 1.286–5.474), higher temperature (AOR:1.485, 95% CI: 1.080–2.042), higher systolic BP (AOR:1.057, 95% CI:1.037–1.078), raised haematocrit (AOR: 1.953, 95% CI: 1.010–3.778), elevated creatinine (AOR:3.504, 95% CI:1.351–9.008) and elevated ALT (AOR: 4.146, 95% CI:1.878–9.154). Obesity was found to be significantly associated with hospitalization >3 days (AOR: 1.990, 95% CI: 1.134–3.494) and the presence of increasing haematocrit with decreasing platelets (AOR: 2.134, 95% CI = 1.235–3.688). Serial assessment of laboratory data revealed that peak haematocrit was significantly higher and nadir platelets levels were significantly lower in obese patients. Both peak and admission levels of leukocyte counts, AST, ALT and creatinine were significantly higher in the obese group. Conversely, both admission and nadir albumin levels were lower for the obese group, although only nadir albumin levels achieved statistical significance. These findings support closer clinical monitoring of obese patients who present with DENV infections, as this patient cohort may possess an increased tendency towards developing more severe clinical manifestations of DENV infections as compared to non-obese patients.

## Introduction

Dengue virus (DENV) infections caused by DENV serotypes -1 to -4, have gained notoriety as one of the most important mosquito- borne viral infections in the world [1, 2]. Transmitted predominantly by the *Aedes aegypti* mosquito, dengue has emerged as a major public health threat in the tropical and subtropical regions of the world and continues to spread at an alarming rate [1, 2]. Current data estimates that nearly 400 million DENV infections occur yearly worldwide, of which 25% are clinically apparent [1]. Nearly 75% of the global population at risk for DENV infections reside in the Asia-Pacific region [1]. Malaysia is hyperendemic for dengue and has been experiencing an exponential rise in dengue cases, with a sharp increase occurring between 2013 to 2014 (43,346 cases to 108, 698 cases). In 2016 alone 101, 357 dengue cases, with 237 deaths were reported [3].

DENV infections were previously classified as dengue fever (DF), dengue haemorrhagic fever (DHF) and dengue shock syndrome (DSS). The revised World Health Organization (WHO) dengue classification of 2009 represents a more practical criterion that utilizes the presence of dengue warning signs to improve the ability to detect infections that that may progress to severe dengue [4]. While a large proportion of patients undergo a mild self-limiting clinical course, a smaller percentage develop more severe manifestations; which ranges from haemorrhagic manifestations to life threatening shock syndrome, multi-organ impairment and death [1, 4]. In addition to the various virologic factors, several host and clinical factors thought to confer risk of developing more severe form of dengue have been investigated, including host immune status, genetic susceptibility, gender, age, pregnancy and the presence of comorbidities [2, 5].

Nutritional status may have a role in determining the severity of DENV infection although previous studies have shown controversial outcomes [6]. Between the two extremes of malnutrition and obesity, the latter represents a growing problem in developing countries such as Malaysia. Obesity and overweight have been steadily increasing in the Southeast Asian countries over the past three decades, driven by interlinked factors such as rising incomes, urbanisation, shifting lifestyles and genetic factors [7]. The 2015 National Health and Morbidity Surveys (NHMSs) reported that 30.0% and 17.7% of the Malaysian adults were overweight and obese respectively [8], making Malaysia the country with the highest obesity prevalence in Southeast Asia [7].

Obesity can profoundly influence normal physiology of the human body and a dynamic interaction between adipose tissue, inflammatory response, immune system and infections has been uncovered [9–11]. Recent research has linked obesity to immune dysfunction, making obese individuals more susceptible to various infections [9–11]. Leptin is a major mediator of the altered immune balance in the obese individuals and has been shown to promote macrophage phagocytosis, increase secretion of pro-inflammatory cytokines and modulate the adaptive immune system [9]. Immune cell dysfunction because of obese conditions may result in impaired host defence which may predispose patients to nosocomial, periodontal, respiratory, hepatobiliary, gastrointestinal and postoperative infections [9–11]. The association between obesity and worse clinical outcomes has been well established in the 2009 influenza A (H1N1) pandemic strain [10, 12]; as infected obese patients were at greater risk of ICU admissions and deaths and had longer ICU and hospital stays compared to the non-obese patients [12]. Subsequent studies on obese mice showed that high leptin levels may be an important mediator for severe pulmonary inflammatory damage in these patients [13]. Besides, obese individuals are hyperleptinemic and possess high levels of SOCS3, a protein regulator of leptin levels [14]. Elevated leptin and SOCS3 levels correlates with a decreased type 1 interferon response, which serves as a crucial innate immune system activator in stimulating an antiviral state [9].

Studies investigating the relationship between nutritional status and dengue severity are scarce. Moreover, these studies focus mainly on children and the evidence linking obesity and the various clinic-pathological characteristics of DENV infection have been inconsistent [6, 15–17]. Despite an increasing prevalence of both obesity and DENV infections, there persists a lack of information linking these two entities. Another important distinction regarding this study compared to previous studies is that it reports in detail the various clinical and laboratory findings both on admission and during serial monitoring. This exhaustive exploration is integral towards better understanding of the clinicopathological correlates of DENV infection and obesity.

The body-mass index (BMI) is an easily obtainable clinical parameter and would serve as a convenient, accessible and low-cost means of prognosticating the outcome of DENV infections, along with other clinicopathological markers. Given the high prevalence of both obesity and dengue in Malaysia, as well as the unpredictable nature of the clinical manifestations of DENV infections, we sought to investigate the influence of obesity on various clinical and laboratory parameters amongst DENV infected patients.

## Materials and methods

This research was conducted at Hospital Sultanah Aminah Johor Bahru (HSAJB) which is the main tertiary referral center in southern Malaysia. It has over 1000 beds, with its patient population reflective of a larger community in Malaysia. During the study period (April- July 2015), hospitalized patients aged > 12 years who were positive for non-structural protein 1 (NS1) dengue antigen were included in this study. Testing was done using a commercially available rapid dengue diagnostic kit, the SD BIOLINE Dengue Duo combo device (Standard Diagnostic Inc., Korea).

Clinical data was collected retrospectively from the medical case notes, microbiology, haematology and biochemical laboratory charts. The information retrieved on admission included demographic data, vital signs, height, weight, underlying comorbidities, signs and symptoms, haematological and biochemical parameters. As part of the standard protocol for dengue management, routine serial haematological and biochemical tests were performed, and relevant nadir or peak levels were recorded. Warning signs and severe dengue manifestations were documented throughout the duration of hospital stay.

This study was approved by the Medical Research Ethics Committee, Ministry of Health Malaysia (NMRR-15-1543-27545). As this was a retrospective study, informed consent was not obtained from the patients and information was analyzed anonymously.

### Definition

Obesity for adults was defined as body mass index (BMI) of 27.5 kg/m^2^ or higher [18]. The cut-off values were based on WHO-suggested classifications for Asian populations and is also used by our local Malaysian Obesity Clinical Practice Guideline [18, 19]. For adolescents aged between 12 to 18 years of age in our study, BMI-for-age charts were used to define their BMI grouping based on the National Center for Health Care Statistics and the Centers for Disease Control (CDC) reference standards. (https://www.cdc.gov/growthcharts/cdc_charts.htm).

Dengue warning signs (WS) comprised of persistent vomiting (≥ 2 consecutive days), persistent diarrhea (passage of ≥ 3 loose stools per day), abdominal pain or tenderness, clinical fluid accumulation, hepatomegaly (>2 cm) and haematocrit rise concurrent with a rapid decrease in platelet counts [4, 20].

Severe dengue was defined as per WHO 2009 guidelines [4] with minor modifications [21, 22] and included at least one of the 3 criteria:

1. Severe plasma leakage leading to shock or fluid accumulation with respiratory distress (respiratory rate ≥30/min with oxygen saturation ≤ 92% on room air, or requiring mechanical ventilation) [4, 21].
2. Severe bleeding- bleeding with hemodynamic instability necessitating fluid replacement for shock and/or whole blood or packed cell transfusion or any life-threatening bleed, e.g. haematemesis, melaena or intracranial bleed [4, 21].
3. Severe organ impairment comprised severe liver impairment (aspartate aminotransferase or alanine aminotransferase ≥1000 IU/L), encephalopathy, myocarditis or acute renal impairment (Stage 2 Acute Kidney Injury) [4, 22].

Major haemorrhage referred to clinically significant bleeding requiring red cell transfusion whereas, minor haemorrhage referred to mucosal bleeding such as gum bleeding, nasal bleeding not requiring red cell transfusion [23]. Severe thrombocytopenia was defined as platelet count < 50,000/ mm^3^, a level shown to be associated with additional severe manifestations [22].

We compared the differences in epidemiological, clinical and laboratory measures between patients who were obese and those who were not. Data was analyzed using IBM^®^ SPSS^®^ Statistics 23.0. Categorical variables were expressed as numbers and percentages, while median and range were used to describe the continuous variables. Kolmogorov-Smirnov test was used to determine the normality of continuous data. Associations between obesity and demographic characteristics, initial clinical presentation, co-morbidities and laboratory data on admission, as well as clinical characteristics based on disease severity were initially determined with binary logistic regression. Multivariate stepwise backward logistic regression analysis was subsequently performed by including parameters with *p*<0.2 in univariate analysis to determine the independent factors associated with obesity. Model fitness was determined with the Hosmer-Lemeshow test. Variables with more than 5% of missing data were excluded from the final model to increase reliability of results. Laboratory findings on admission and serial assessment were also compared between obese and non-obese groups using Mann Whitney U test.

## Results

During the study period, 383 patients with NS1 antigen positivity (age >12 years) required hospitalization. Medical notes for 355 patients were available for analysis (not traceable; n = 16; incomplete data; n = 12). Records of height and weight were available for 335 patients and these patients were included in our study. There were 190 (56.7%) male and 145 (43.3%) female patients with a median age of 30.2 years (range, 12.3 to 73.2 years). Majority of the patients were Malays (48.1%) followed by Chinese (20%), and Indians (13.7%), which parallels closely the ethnic makeup of the Malaysian population. Based on the above BMI classification, 10.7% (n = 36) patients were underweight, 43.3% (n = 145) normal-weight, 24.5% (n = 82) pre-obese, 17.9% (n = 60) obese class I, 2.7% (n = 9) obese class II and 0.9% (n = 3) obese class III; i.e. 72 patients (21.5%) were in the obese category.

### The association of obesity and clinic-pathological characteristics on admission

Table 1 illustrates the association between obesity and the various clinical and laboratory finding on admission.

**Table 1:** Demography, initial clinical presentation, co-morbidities and laboratory data on admission in patients with and without obesity.

		All patients (n = 335)	Obese group (n = 72)	Non-obese group (n = 263)	OR	95% CI	*P*	AOR^f^	95% CI	*P*
*Demography*	Age (years)	30.2 (12.3–73.2)	32.9 (12.6–64.2)	29.3 (12.3–73.2)	1.012	0.994–1.031	0.203			
	Females	145(43.3)	33(45.8)	112(42.6)	1.141	0.675–1.927	0.622			
*Initial symptoms / signs*	Days of fever	4(1–14)	4(1–14)	4(1–8)	1.006	0.835–1.211	0.952			
	Chills and rigors	71(21.2)	22(30.6)	49(18.6)	1.922	1.065–3.466	0.028	2.653	1.286–5.474	0.008
	Headache	135(40.3)	34(47.2)	101(38.4)	1.435	0.849–2.426	0.176			
	Upper respiratory tract symptoms	53(15.8)	13(18.1)	40(15.2)	1.228	0.617–2.445	0.558			
	Vomiting	233(69.6)	48(66.7)	185(70.3)	0.843	0.483–1.472	0.548			
	Diarrhoea	180(53.7)	43(59.7)	137(52.1)	1.364	0.803–2.316	0.250			
	Abdominal pain	165(49.3)	33(45.8)	132(50.2)	0.840	0.498–1.416	0.512			
	Any musculoskeletal symptoms^a^	196(58.5)	48(66.7)	148(56.3)	1.554	0.899–2.686	0.113			
	Rash	50(14.9)	11(15.3)	39(14.8)	1.036	0.501–2.142	0.925			
	Neurological symptoms	30(9)	7(9.7)	23(8.7)	1.124	0.462–2.735	0.797			
	Haemorrhagic symptoms	36(10.7)	5(6.9)	31(11.8)	0.558	0.209–1.492	0.240			
	Temperature (°C)	37.8(36–40)	38(36–40)	37.8(36–40)	1.324	1.012–1.732	0.041	1.485	1.080–2.042	0.015
	Respiratory rate/min	18(12–36)	18(15–21)	18(12–36)	0.852	0.721–1.006	0.059			
	Pulse rate/min	93(45–136)	95(45–132)	92(52–136)	1.009	0.992–1.026	0.288			
	Oxygen saturation (%)	99(87–100)	99(87–100)	99(94–100)	0.903	0.764–1.067	0.230			
	Systolic blood pressure (mmHg)	120(81–202)	133(94–168)	117(81–202)	1.049	1.032–1.067	<0.001	1.057	1.037–1.078	< 0.001
	Diastolic blood pressure (mmHg)	73(35–113)	78(48–99)	71(35–113)	1.042	1.018–1.066	<0.001			
*Comorbidities*	Any comorbidities^b^	52(15.5)	14(19.4)	38(14.4)	1.429	0.726–2.814	0.300			
	Diabetes mellitus	13(3.9)	3(4.2)	10(3.8)	1.100	0.295–4.107	1.000			
	Hypertension	28(8.4)	11(15.3)	17(6.5)	2.609	1.162–5.857	0.017			
	Respiratory condition	8(2.4)	2(2.8)	6(2.3)	1.224	0.242–6.196	0.683			
	Ischaemic heart disease	5(1.5)	1(1.4)	4(1.5)	0.912	0.100–8.288	1.000			
	Immunosuppressive conditions^c^	8(2.4)	1(1.4)	7(2.7)	0.515	0.062–4.256	1.000			
*Initial laboratory data*	Raised haematocrit^d^	182(54.3)	45(62.5)	137(52.1)	1.533	0.898–2.617	0.116	1.953	1.010–3.778	0.047
	Leukopenia	270(80.6)	53(73.6)	217(82.5)	0.591	0.320–1.092	0.091			
	Platelet < 50 (x10^9^/L)	79(23.6)	14(19.4)	65(24.7)	0.735	0.385–1.405	0.351			
	Platelet < 100 (x10^9^/L)	213(63.6)	46(63.9)	167(63.5)	1.017	0.591–1.750	0.951			
	Elevated urea^e^	9 (2.8)	3(4.3)	6(2.3)	1.909	0.465–7.836	0.405			
	Low sodium (mmol/L)^e^	23(7.0)	7(10.1)	16(6.2)	1.708	0.673–4.332	0.288			
	Low potassium (mmol/L)^e^	219(66.8)	46(66.7)	173(66.8)	0.984	0.566–1.746	0.994			
	High creatinine (μmol/L)^e^	30(9.2)	13(18.8)	17(6.6)	3.291	1.511–7.168	0.002	3.504	1.351–9.008	0.01
	High bilirubin (μmol/L)^e^	14(4.3)	4(5.8)	10(3.9)	1.526	0.464–5.023	0.504			
	Low albumin (g/L)^e^	7(2.1)	1(1.4)	6(2.3)	0.620	0.073–5.238	1.000			
	High ALT (IU/L) ^e^	200(61)	55(79.7)	145(56)	3.089	1.635–5.834	<0.001	4.146	1.878–9.154	<0.001
	High AST (IU/L^)^ ^e^	236(83.7)	54(88.5)	182(82.4)	1.653	0.700–3.906	0.248			
	High creatinine kinase (IU/L) ^e^	138(50.5)	40(66.7)	98(46)	2.347	1.287–4.279	0.005			
	High LDH (IU/L) ^e^	226(83.4)	52(89.7)	174(81.7)	1.943	0.779–4.843	0.148			
	Ig G	80(31.6)	24(37.5)	56(29.6)	1.425	0.786–2.583	0.242			

Note: Data presented as n (%) for categorical variables and median (range) for continuous variables

^a^Any musculoskeletal symptoms refer to myalgia, arthralgia and bone pain

^b^ A patient may have more than one comorbidities.

^c^Immunosuppressive conditions include HIV (n = 2), connective tissue disease (n = 3), steroid intake (n = 2) and malignancy (n = 2)

^d^Haematocrit categorization on admission was based on a population background study conducted in Malaysia: >40% in female adults (all age group), > 46% in male ≤ 60 years, > 42% in male > 60 years and > 38% in children aged up to 12+ [20]

^e^The denominator is not 335 and % have been adjusted accordingly.

^f^Multivariate stepwise backward logistic regression was performed. Model fitness was tested with Hosmer-Lemeshow test (p = 0.153). Final model has an overall correct classification of 86.7%.

There were no significant differences between the obese and non-obese patients in terms of age and gender distribution. At least one preexisting comorbidity was present in 15.5% patients. Hypertension was the commonest comorbidity, affecting 8.4% of patients and was more prevalent among the obese patients. Median duration of symptoms before hospitalization was 4.0 days (range 1–14 days) with no significant differences between the obese and non-obese group. Most frequently reported symptoms on admission were vomiting (70%), musculoskeletal symptoms (58.5%) and diarrhea (53.7%). On admission, hematological derangements were common, whereby 54.3% of patients had raised haematocrit, 63.6% had thrombocytopenia (with 23.6% having platelets < 50,000/ mm^3^) and 80.6% had leukopenia (< 4 x 10^9^/L). Elevated AST and ALT were detected in 83.7% and 61.7% patients respectively, whereas 83.4% patients had raised lactate dehydrogenase (LDH) and 50.5% had raised creatinine kinase (CK) respectively.

Univariate analysis of the comparison of clinic-pathological findings upon admission between patients with and without obesity is shown in Table 1. The following admission clinical and laboratory parameters were significantly associated (p < 0.05) with obesity in univariate analysis: chills and rigors, higher temperature, higher systolic and diastolic blood pressure, elevated creatinine, ALT and creatinine kinase were more frequently observed in obese as compared to non-obese patients.

Multivariate analysis revealed that elevated ALT was associated with the highest likelihood of association with obesity (AOR: 4.146, 95% CI:1.878–9.154, p<0.001), followed by elevated creatinine level (AOR: AOR:3.504, 95% CI:1.351–9.008, p = 0.01), occurrence of chills and rigors (AOR:2.653, 95% CI: 1.286–5.474, p = 0.008), raised haematocrit (AOR: 1.953, 95% CI: 1.010–3.778, p = 0.047), body temperature (AOR:1.485, 95% CI: 1.080–2.042, p = 0.015) and systolic blood pressure (AOR:1.057, 95% CI:1.037–1.078, p<0.001). Although elevated creatinine kinase and LDH levels had p<0.2 in univariate analysis, it was not subjected to multivariate analysis, as missing values were >5%. The final multivariate model was fit and 86.7% of cases were correctly classified.

### Comparison between admission and peak/nadir levels of laboratory parameters

Comparisons of laboratory parameters between obese and non-obese groups at admission and upon serial assessment are presented in Table 2.

**Table 2:** Laboratory findings on admission and on serial assessment in patients with and without obesity.

	Admission				Peak / Nadir			
	All patients (n = 335)	Obese group (n = 72)	Non-obese group (n = 263)	*P* ^a^	All patients (n = 335)	Obese group (n = 72)	Non-obese group (n = 263)	*P* ^a^
Haematocrit (%)	43.3(21–67.7)	44(26–54.2)	43(21–67.7)	0.244	45(25–67.7) ^b^	47(32–56)	45(25–67.7)	0.014*
Platelet count (x10^9^/L)	81(4–433)	80.5(4–242)	81(7–433)	0.584	43.5 (1–300) ^c^	32(1–191)	48(2–300)	0.014*
Leukocyte count (x 10^9^/L)	3(0.9–1.810)	3.75(1.7–11.5)	2.86(0.9–18.1)	0.001**	2.2(0.7–11.2) ^c^	2.6(1.1–10.1)	2.1(0.7–11.2)	<0.001**
Creatinine (μmol/L)	71(31–587)	80(44–587)	70(31–250)	0.002*	78(36–592) ^b^	84(50–592)	77(36–385)	0.005*
Albumin (g/L)	40(12–62)	39(27–48)	40(12–62)	0.080	37(12–44) ^c^	35(28–43)	37(12–44)	0.050*
ALT (IU/L)	53(4–1666)	79(10–1666)	45(4–644)	<0.001**	95.5(8–1666) ^b^	132(25–1666)	89(8–754)	0.012*
AST (IU/L)	91(15–1344)	142.5(21–1344)	86(15–1297)	0.008*	164(22–1860)^b^	213(22–1344)	154(23–1860)	0.028*
Creatinine kinase (IU/L)	199(15–4432)	267(55–3227)	156(15–4432)	0.121	263(33–8098)^b^	354(76–8098)	238(33–6553)	0.172
LDH (IU/L)	378.5(108–2789)	445(171–2789)	358(108–1681)	0.635	498(159–2789)^b^	684(195–2789)	478(159–2492)	0.004*

**Note:** Data presented as median (range)

^a^ Mann Whitney U test was applied.

^b^ peak levels,

^c^ nadir levels

*significant at p<0.05;

**significant at p<0.001

Although there were no statistically significant differences in the median haematocrit and platelet levels on admission between the two groups, peak haematocrit was significantly higher and nadir platelets levels were significantly lower in the obese group. The median levels of leukocyte count, creatinine, AST and ALT levels were significantly higher in the obese group for both the admission as well as the peak levels. Both admission and nadir albumin levels were lower for the obese group, although it attained statistically significant difference only for the nadir levels. Compared to the non-obese group, peak levels for LDH were significantly higher in the obese group.

### The impact of obesity on disease severity

The comparison of disease severity between obese and non-obese patients is shown in Table 3.

**Table 3:** Association between clinical characteristics based on disease severity^a^ in patients with and without obesity.

	All patients (n = 335)	Obese group (n = 72)	Non-obese group (n = 263)	OR	95% CI	*P*	AOR^b^	95% CI	*P*
Any warning signs^c^	301(89.9)	66(91.7)	235(89.4)	1.311	0.521–3.299	0.565			
Persistent vomiting	151(45.1)	36(50)	115(43.7)	1.287	0.763–2.170	0.343			
Persistent diarrhoea	128(38.2)	31(43.1)	97(36.9)	1.294	0.762–2.197	0.339			
Abdominal pain/ tenderness	164(49)	36(50)	128(48.7)	1.055	0.626–1.777	0.841			
Restlessness & confusion	18(5.4)	6(8.3)	12(4.6)	1.902	0.688–5.256	0.237			
Mucosal bleeding	52(15.5)	11(15.3)	41(15.6)	0.976	0.474–2.013	0.948			
Tender hepatomegaly	6(1.8)	0	6(2.3)	NA	NA	0.347			
Pleural effusion	5(1.5)	2(2.8)	3(1.1)	2.476	0.406–15.109	0.293			
Increasing haematocrit with decreasing platelets	150(44.8)	41(56.9)	109(41.4)	1.869	1.103–3.165	0.019*	2.134	1.235–3.688	0.007*
Any severe dengue manifestations^d^	29(8.7)	7(9.7)	22(8.4)	1.180	0.483–2.883	0.717			
Fluid accumulation with respiratory distress	6(1.8)	3(4.2)	3(1.1)	3.768	0.744–19.082	0.116			
Shock	7(2.1)	0	7(2.7)	NA	NA	0.353			
Severe bleeding	5(1.5)	1(1.4)	4(1.5)	0.912	0.100–8.288	1.000			
Severe organ involvement	17(5.1)	4(5.6)	13(4.9)	1.131	0.357–3.581	0.768			
Mortality	2(0.6)	1(1,4)	1(0.4)	3.783	0.234–61,245	0.378			
Hospitalization > 3 days	177(52.8)	46(63.9)	131(49.8)	1.783	1.041–3.054	0.034*	1.990	1.134–3.494	0.017*
Duration of admission	4(0–13)	4(2–10)	3(0–13)	1.133	0.961–1.335	0.138			
Minor haemorrhages	23(6.9)	4(5.6)	19(7.2)	0.755	0.249–2.295	0.795			
Major haemorrhage	17(5.1)	2(2.8)	15(5.7)	0.472	0.106–2.115	0.543			
Nadir platelet < 50 (x10^9^/L)	177(54.1)	47(65.3)	130(51)	1.808	1.049–3.114	0.032*			
LDH > 600 (IU/L)	82(30.1)	29(50)	53(24.8)	3.038	1.665–5.541	<0.001**			
Liver enzymes> 400 (IU/L)	39(11.9)	12(17.1)	27(10.4)	1.778	0.850–3.720	0.123			
Intravenous fluid	322(96.1)	69(95.8)	253(96.2)	0.909	0.243–3.394	1.000			
ICU admission	10(3)	3(4.2)	7(2.7)	1.590	0.401–6.310	0.453			
Oxygen	3(0.9)	1(1.4)	2(0.8)	1.838	0.164–20.562	0.517			
Mechanical ventilation	3(0.9)	1(1.4)	2(0.8)	1.838	0.164–20.562	0.517			
Inotropes	3(0.9)	1(1.4)	2(0.8)	1.838	0.164–20.562	0.517			

Data presented as n (%)

^a^Disease severity is based on WHO (2009) classification plus additional criteria

^b^Multivariate stepwise backward logistic regression was performed. Model fitness was tested with Hosmer-Lemeshow test (p = 0.903). Final model has an overall correct classification of 78.3%.

^c^A patient may have more than one warning signs

^d^A patient may have more than one severe dengue manifestations

NA- Not applicable

*significant at p<0.05;

**significant at p<0.001

The median (range) length of hospital stay was 3 (range 0–13) days for the non-obese and 4 (range 2–10) days for the obese group. Patient who were obese had a higher frequency of hospitalization of longer than 3 days on univariate analysis (p < 0.05).

Overall, 90% of patients presented with at least one warning sign. Abdominal pain/tenderness (49%), persistent vomiting (45.1%) and increasing haematocrit with decreasing platelets (44.8%) were the most common dengue warning signs. However, increasing haematocrit with decreasing platelets which was more frequent in the obese patients (56.9%) compared to those who were non-obese (41.4%) was the only dengue warning sign that achieved statistical significance (p < 0.05).

In total, 29 patients (8.7%) presented with severe dengue manifestations (WHO 2009 classification): these were fluid accumulation with respiratory distress (n = 6), shock (n = 7), severe bleeding (n = 5), and severe organ impairment (n = 17). The organs involved were the liver (n = 12), kidneys (n = 6) and central nervous system (n = 1). However, we found no statistical differences between the two groups for the listed severe dengue manifestations, haemorrhagic manifestation and the management of the patients in terms of ICU admission and other interventions. Severe thrombocytopaenia (nadir platelet < 50 x10^9^/L) and LDH > 600 (IU/L) was significantly more common amongst the obese group in univariate analysis.

The multivariate analysis revealed obese patients to be significantly associated with hospitalization of more than 3 days (AOR: 1.990, 95% CI: 1.134–3.494, p = 0.017) and the presence of increasing haematocrit with decreasing platelets (AOR: 2.134, 95% CI = 1.235–3.688, p = 0.007). Although LDH > 600 IU/L was significantly higher in the obese group in univariate analysis, it was not subjected to multivariate analysis, as missing values were >5%. The final multivariate model was fit and 78.3% of cases were correctly classified. The associations in the multivariate analysis were maintained after being adjusted for comorbidity and age.

## Discussion

With an upsurge of both obesity and dengue cases in Malaysia [3, 8], this study presented us with a unique opportunity to explore the association between obesity and the various clinical and laboratory parameters in DENV infected patients. In a large study it was revealed that obese children were more susceptible to acquiring DENV infection compared to non-obese children [15]. Adipocytes and adipose tissue macrophages have been identified as potential targets for DENV infection and production [24], a plausible explanation to this association.

To our best knowledge, this study is the first to specifically explore the association between obesity and DENV infection amongst predominantly adult patients (92.5%).

Our data suggests that obese patients had higher frequency of raised haematocrit on admission. Moreover, peak haematocrit levels were significantly higher in the obese group, although there was no statistically significance difference in the admission haematocrit levels between the two groups. Vascular endothelial dysfunction leading to plasma leakage is a well-known feature of DENV infection, as evidenced by a recent study linking elevated levels of syndecan-1, chondroitin sulfate (components of endothelial glycocalyx layer), and claudin-5 (component of interendothelial junction) with severe plasma leakage in dengue infections [25]. Furthermore, various proinflammatory and angiogenic factors such as TNF-α, IL-8, VEGF, VEGFRs and angiopoietin-1 and -2, were found to be upregulated in DENV infection [26]. Notably, vascular disease in the obese have been credited to endothelial dysfunction that occurs because of chronic pro-inflammatory environment [27]. Taken together, it is possible that DENV infections may further damage the already dysfunctional endothelium of obese patients, leading to further plasma leakage and subsequently higher haematocrit levels. Other markers of plasma leakage such as lower albumin levels, pleural effusion and fluid accumulation with respiratory distress were higher amongst the obese patients, although statistical significance was attained only for lower albumin levels. Contrary to our finding, in another large study involving children, the frequency of raised haematocrit and low albumin did not differ between patients with different nutritional status [15].

Bone marrow suppression and platelet destruction have been hypothesized as the cause of thrombocytopenia in DENV infections, with a rapid decrease in platelet counts constituting a WHO dengue warning sign [4, 23, 28]. Platelet levels generally show a dynamic decline, reaching a nadir by 3 to 6 days of fever onset [23]. Contrary to the findings of Kalayanarooj et al.[15], obese patients in our study were found to have statistically lower nadir platelet levels, although the admission platelets levels between the two groups were not significantly different. In vitro evidence suggests that there is an increased adherence of platelets to damaged vascular endothelium infected by DENV, contributing to thrombocytopaenia [28]. As the obese patients have pre-existing vascular endothelial dysfunction [27], the additional vascular damage caused by DENV infection may trigger an accelerated rate of platelet adherence, contributing to lower nadir platelet levels [28]. Despite having lower nadir platelets, the risk of minor and major haemorrhages was not higher, suggesting that other factors such as hepatic derangement, coagulopathy, vasculopathy and thrombocytopenia act synergistically to cause bleeding [23, 28].

Plasma leakage and thrombocytopaenia are the major pathophysiologic hallmarks in DENV infections and are indices that can be used to assess disease severity [25, 26]. Our study showed that amongst the warning signs, the frequency of patients with haematocrit rise concurrent with a rapid decrease in platelet counts was significantly higher amongst the obese compared to non-obese patients. Taken together, our observations are in line with Pichainarong et al. [17] and suggests obesity contributes to a more severe form of dengue.

The association between nutritional status of children and the severity of dengue disease has been reported in several studies, with inconclusive results [6]. Some findings show obese children are at increased risk of developing severe forms of dengue haemorrhagic fever (DHF) and dengue shock syndrome (DSS) [17, 29]. Emerging evidence suggests that obesity is associated with chronic, low-grade systemic inflammation, with adipose tissue seen as a secretory organ rather than an inert tissue. Circulating macrophages have been documented to infiltrate white adipose tissue, with the number of macrophages directly correlating to the size of adipocytes and adiposity. These macrophages are significant source of inflammatory cytokines such as TNF-α and IL-6 [30]. At the same time, these pro-inflammatory adipokines have been implicated in causing endothelial dysfunction seen in DENV infections [9, 27]. DENV infections in obese patients may result in an overwhelming production of cytokines, driving the development of more severe disease. In line with these propositions, malnutrition, a condition marked by decreased cellular immune response, has been found to be a protective factor against DENV infections in malnourished children [31, 32]. Conversely, in other studies, overweight/obesity did not appear to be a risk factor for dengue severity [6, 16], neither did malnutrition appear to be predictive of good outcomes [15, 16]. Yet other studies [6, 15] reported that malnourished children were at a greater risk of DSS compared to normal and obese children, attributing this to smaller volume of extra-cellular fluid and plasma volume in the malnourished patients, with shock developing with lesser degree of plasma leakage [15]. These inconsistent results highlight the need for further investigations examining the relationship between nutritional status and outcomes in DENV infection.

Our findings showed that obese patients had significantly higher admission as well as peak creatinine levels. The mechanisms linking obesity and elevated creatinine are multifactorial. Obesity is a state of excess adiposity and is widely recognized as a risk factor for diabetes, hypertension, cardiovascular diseases [9], conditions that can lead to renal impairment. All these conditions were found in a higher proportion in our obese cohort, although only the presence of hypertension attained statistical significance. Moreover, increased secretion of adipokines such as proinflammatory cytokines like TNFα, IL-6 and leptin can result in endothelial dysfunction [9, 27], with the possible involvement of the of kidney vasculature. In more severe DENV infections, acute kidney injury (and by extension, raised serum creatinine) is a potential complication.

Liver damage is a well-established feature of DENV infected patients and higher liver enzyme levels have been associated with increasing disease severity [33]. Pathological investigations of DENV-infected livers reveal a range of cellular abnormalities including steatosis and hepatocellular necrosis [33]. These findings suggest that higher liver enzyme levels found in more clinically severe DENV infections may indicate increased hepatocyte destruction. Consistent with this and other reports [15], significantly higher number of obese patients had elevated ALT during admission, as well as higher admission and peak levels of ALT and AST. The deranged liver function in obese patients may be compounded by hepatic non-alcoholic steatosis fatty liver disease [34], which is not an uncommon finding in DENV infections. In fact, studies have shown as association between lipid droplets alteration and DENV viral replication, which contributes to the spread of the virus in hepatocytes [34, 35].

LDH is an enzyme expressed ubiquitously in the body, including liver and muscle cells. Cell injury or death of these cells is marked by an increase in serum LDH as this enzyme is released into the circulation [36]. Increased LDH levels have previously been documented to be an indicator of severity in DENV infection, and may likely reflect muscle and liver cell injury [37]. In our study, 83.4% patients were found to have elevated LDH levels. Interestingly, 50% of our obese cohort had levels more than 600 IU/L, compared to only 24.8% of our non-obese group (P<0.001), a level associated with severity of dengue [37]. It is interesting to note that a study conducted amongst apparently healthy adults showed that total body fat was associated with elevated hepatic enzymes as well as LDH [38], thus, DENV infection possibly accentuates these levels.

Leukopenia is a common feature in DENV infections, with 80% of our patients showing leukopenia upon admission. However, in our study, obese patients had significantly higher admission and peak leukocyte counts and had lower frequency of patients with leukopenia on admission. A plausible hypothesis is that adipokines such as leptin might be involved in increased leukocyte counts in obese patients, as leptin has been reported to stimulate myeloid differentiation from human bone marrow haematopoietic progenitors [39]. Significantly higher frequency of chills and rigors and higher temperatures were recorded amongst obese patients compared to the non-obese patients. This may be due to the poor heat-dissipating properties of fatty tissue, leading to dysfunctional thermoregulatory mechanisms in the obese [40]. While some studies revealed that hepatomegaly was less common while maculopapular rash and encephalopathy were found more often in obese patients [15], our study showed no such association. The duration of hospital stay was significantly longer in the obese patients, a probable indicator for illness severity and comorbid conditions seen in these patients. Although we were not able to determine the link between obesity and secondary DENV infection and DENV serotypes amongst our patients, no link between primary and secondary DENV infections and nutritional status was reported by Kalayanarooj S et al. [15]. Similarly, there was no association between different nutritional status and DENV serotypes [15].

There are several limitations to this current study. All retrospective studies depend on the obtainability, accurateness and comprehensiveness of medical data. In this study, the presence or absence of symptoms was dependent on a written documentation in the patients’ notes with non-documentation interpreted as the absence of the symptoms, although it is also likely that the symptom was not assessed for. However, despite this, all DENV infected patients in Malaysia are managed with a standardized algorithm which includes the careful recording of various clinical and laboratory parameters which have been pre-determined to vital for patient monitoring. Strict compliance to the algorithm has greatly improved the comprehensiveness of data collected.

This study also possesses a relatively small number of patients who developed pleural effusion which may result in a low statistical power to detect a true association of this symptom with obesity. Pleural effusion and ascites may not be evident clinically at early stages or may be overlooked should the volumes be small. However, unlike monitoring parameters such as haematocrit, hepatic transaminases and platelet count, that are routinely done as part of the protocol of management of dengue patients, lateral chest decubitus X-ray film and ultrasonography of the chest/abdomen are not done routinely in Malaysia due to financial and service restraints and are performed at the discretion of the attending clinicians.

Determining the type of DENV infection (primary versus secondary) would have provided valuable added information. Although we reported results of indirect IgG, (which in the presence of a positive NS1 antigen favours secondary DENV infection), categorization of secondary infections based on the results of Panbio dengue IgG capture ELISA would have provided more reliable results.

In summary, this study suggests that DENV infected obese patients possess many clinical parameters suggestive of more severe clinical manifestations as evidenced by a higher frequency of haemoconcentration, severe thrombocytopenia, elevations of creatinine, liver enzymes, warning sign of increasing haematocrit with rapid drop in platelets and longer duration of hospital stay. In view of the greater disease severity associated with DENV infections amongst obese patients, heightened vigilance is needed when treating these group of patients.
